# Supplementary material for: Live-Cell Imaging of Single Neurotrophin Receptor Molecules on Human Neurons in Alzheimer’s Disease
Source: Int J Mol Sci. 2021 Dec 9;22(24):13260. doi: 10.3390/ijms222413260 (PMC8708879; doi:10.3390/ijms222413260)
Supplement: Supplementary file 1 [file ijms-22-13260-s001.zip › ijms-1474203-supplementary.pdf]

Supplementary materials

Supplementary methods

All chemicals were purchased from MERCK (Sigma-Aldrich; St Louis, MO, USA), whereas the cell culture reagents and culture plates were obtained from Thermo Fisher Scientific (Waltham, MA, USA), unless specified otherwise.

Generation of hiPSC-derived neurons

The hiPSC lines were maintained on Matrigel (BD Matrigel; CatN: 07181; Stem Cell Technologies) in mTESR1 (CatN: 85850; Stem Cell Technologies, Vancouver, Canada) culture media. The media was changed daily, and the cells were passaged every 5-7 days using Gentle Cell Dissociation Reagent (CatN: 100-0485; Stem Cell Technologies, Vancouver, Canada), according to the manufacturer's instructions.

Neural progenitor cells (NPCs) were generated from each of the hiPSCs by dual inhibition of the SMAD signaling pathway using LDN193189 and SB431542 [1]. Neural induction was initiated upon reaching approx. 90% confluence of iPSCs on Matrigel-coated dishes by addition of Neural Induction Medium (NIM) (1:1 (v/v) mixture of Dulbecco's Modified Eagle's/F12 (CatN: 31331-028) and Neurobasal Medium (CatN: 21103-049), 1x N-2 Supplement (CatN: 17502-048), 1x B-27 Supplement (CatN: 12587010), 1x Nonessential Amino Acids (NEAA; CatN: M7145), 2 mM L-Glutamine (CatN: 35050-038), 50 U/ml Penicillin/Streptomycin (CatN: 15140-122), 100 μM β-mercaptoethanol (CatN: 31350010), 5 μg/ml insulin CatN: I9278), which was supplemented with 5 ng/ml basic fibroblast growth factor (bFGF; CatN: PHG0261), 0.2 μM LDN193189 (CatN: S7507; Selleckchem, UK) and 10 μM SB431542 (CatN: S4317). The NIM medium was changed every day. On day 10, neural rosettes were picked manually and re-plated on poly-L-ornithine/laminin (POL/L; CatN:

L2020; CatN: P4957; 0.003%/3 µg/cm^2) coated dishes and expanded in Neural Maintenance Medium (NMM) (1:1 (v/v) mixture of Dulbecco's Modified Eagle's/F12 and Neurobasal Medium, 1x N-2 Supplement, 1x B-27 Supplement, 1x NEAA, 2 mM L-Glutamine, 50 U/ml Penicillin/Streptomycin), and supplemented with 10 ng/ml epidermal growth factor (EGF; CatN: PHG0314) and 10 ng/ml bFGF.

To generate human neurons, NPCs were plated on the POL/L coated dishes and cultured in differentiation medium (NMM without mitogens). For terminal differentiation into cortical neurons, NPCs were plated on POL/L (0.002%/2 µg/cm^2) at a seeding density of 30.000 cells/cm^2 and 100.000 cells/cm^2 for ELISA experiments with NMM medium. The medium was changed every 3-4 days during the terminal differentiation, until week 7.

Measurement of $A\beta_{1-40}$ and $A\beta_{1-42}$ by ELISA in hiPSC-derived cultures

To examine the Aβ production conditioned media was collected after 4 days of hiPSC culture (without media change) at week 7 from one well of a six-well plate. To prevent protein degradation, 4-(2-Aminomethyl) benzenesulfonyl fluoride hydrochloride (AEBSF; CatN: A8456) was added to the media. Extracellular $A\beta_{1-40}$ and $A\beta_{1-42}$ levels were measured using Human β-Amyloid (1-40) ELISA Kit (CatN: 292-62301; Wako, Richmond, VA, USA) and Human β-Amyloid (1-42) ELISA Kit (CatN: 292-64501; Wako, Richmond, VA, USA), according to the manufacturer's instructions. The secreted Aβ levels determined in pM were normalized to the total protein content (µg) of cell lysate. Total protein concentration was determined using a Pierce BCA Protein Assay Kit (CatN: 23225), where cells were lysed with RIPA Lysis and Extraction Buffer (CatN: 89901) supplemented with Halt™ Protease and Phosphatase Inhibitor Cocktail (CatN: 78446) and Pierce™ Universal Nuclease for Cell Lysis (CatN: 88701). Lysed samples were sonicated and centrifuged at 13.000 rpm to collect the supernatants. The signal was detected with Varioskan Flash Multimode Reader (Thermo Fisher

Scientific, Waltham, MA, USA). As all fAD cell lines bearing *PSEN1* mutations showed significant differences ($p<0.01$) from controls and they were not significantly different from each other, we show the results of pooled data (Supplementary Fig. 1.).

Validation of neuronal differentiation: immunocytochemistry and electrophysiology on hiPSCs-derived neurons

First, single-label immunofluorescence was performed to detect neuronal phenotypes. Expression of microtubule-associated protein 2 (MAP2), beta–III tubulin (TUBB3), vesicular glutamate transporter ½ (VGLUT1/2), glutamate decarboxylase 65/67 (GAD65/67), vesicular acetylcholine transporter (VAChT), TrkA and $p75^{NTR}$ were analyzed in terminally differentiated hiPSCs-derived neurons originated from non-demented individuals and *PSEN1* fAD patients. Cells were fixed with 4% PFA (CatN: P6148) (pH 7.4) for 20 minutes, then were pretreated with TRIS containing 1-3% bovine serum albumin (CatN: A7906) and 0.1-0.3% Triton-X, depending on the detectable target protein. Cells then were incubated in the appropriate primary antibodies (see Table S3) overnight at 4°C or 2 hours at room temperature (RT). To detect the signal, cells were incubated for 60-120 minutes at RT with the appropriate secondary antibodies (see Table S3). Cell nuclei were visualized using Vectashield Mounting Medium with DAPI (1.5 µg/ml; CatN: H-1200-10; Vector Laboratories, Burlingame, CA, USA). MAP2, TUBB3, VGLUT1/2, GAD65/67, VAChT, TrkA and $p75^{NTR}$ positive cells were analyzed under fluorescent microscope equipped with 3D imaging module (Axio Imager system with ApoTome; Carl Zeiss MicroImaging GmbH, Germany) controlled by AxioVision 4.8.1 software (Carl Zeiss, Germany) or under confocal laser scanning microscope (Zeiss LSM710, Germany) (CLSM). Helium-neon laser with 633-nm wavelength was used to excite Alexa-647. The diameter of the pinhole aperture was set to gain an optical thickness of 1 µm.

The omission of the primary antibodies resulted in a complete absence of immunoreactivity (Supplementary Fig.2.).

Electrophysiological properties of terminally differentiated hiPSCs neurons obtained from non-demented control subjects were tested using whole-cell patch-clamp recording. Patch pipettes were pulled from borosilicate glass capillaries with filament (1.5 mm outer diameter and 1.1 inner diameter: Sutter Instruments) with a resistance of 2-3 MΩ. The pipette recording solution contained 10 mM KCl, 130 mM K-gluconate, 1.8 mM NaCl, 0.2 mM EGTA, 10 mM Hepes, 2 mM Na-ATP, (pH 7.3 adjusted with KOH). All recordings were performed at 32 °C with the chamber perfused with oxygenated ACSF containing 2.5 mM KCl, 10 mM Glucose, 126 mM NaCl, 1.25 mM NaH_2PO_4, 2 mM $MgCl_2$, 2 mM $CaCl_2$, 26 mM $NaHCO_3$. Whole-cell recordings were made with an Axopatch 700B amplifier (Molecular Devices) using an upright microscope (Nikon Eclipse FN1, Japan) equipped with differential interference contrast optics (DIC). Cells with access resistance below 20 MΩ were used for analysis. Signals were low pass filtered at 5 kHz and digitized at 20 kHz (Digidata 1550B, Molecular Devices, San Jose, CA, USA). Acquisition and subsequent analysis of the acquired data were performed using Clampex9 and Clampfit software (Axon Instruments, Union City, CA, USA). Traces were plotted using Origin8 software (MicroCal Software, Northampton, MA, USA).

Single-molecule imaging of TrkA and p75^{NTR} molecules in live neurons using total internal reflection fluorescence (TIRF) microscopy

Live-cell immunofluorescent labeling was performed to detect TrkA and p75^{NTR} molecules in the plasma membrane of neurons. Terminally differentiated hiPSCs neurons were grown on Poly-L-ornithine and Laminin (POL/L; 0.002%/3 µg/cm^2) coated 35 mm glass-bottom dishes (P35G-1.0-14-C; MatTek Corporation, Ashland, MA, USA) in NMM medium. Neurons were incubated with ATTO-488 or ATTO-633-labeled antibodies directed against the extracellular

N-terminal domain of either rat p75^{NTR} (1:100, Alomone Labs) or rat TrkA (1:100, Alomone Labs, Israel), respectively at 37 °C for 6 min.

Single-molecule imaging of labeled TrkA and p75^{NTR} molecules was carried out on an Olympus IX81 fiber TIRF microscope equipped with ZDC (Z-drift compensation) stage control, a Plan Apochromat objective (100x, NA 1.45, Olympus, Japan) and a humidified chamber (Supertech, Hungary) heated to 37°C and maintaining 5% CO_2. The 35 mm glass-bottom dish containing neurons was mounted in the humidified chamber of the TIRF microscope immediately after *in vivo* labeling. Diode lasers (Olympus, Japan) were used to excite ATTO-488 at 491 nm and ATTO-633 at 640 nm wavelength and emission was detected above 510 nm and at 650-670 nm emission wavelength range, respectively. The angle of the excitation laser beam was set to reach a 100 nm penetration depth of the evanescent wave. A Hamamatsu 9100-13 electron-multiplying charge-coupled device (EMCCD) camera and Olympus Excellence Pro imaging software were used for image acquisition by TIRF microscopy.

Each control and fAD samples were measured in duplicate on average. Experiments were performed for 60 minutes. As the average diffusion coefficient of TrkA and p75^{NTR} in control neurons did not change with time during the 60 minutes measurements (Fig. 3. D) all data collected for each parameter were analyzed together. During the measurement period of ATTO-488-p75^{NTR} and ATTO-633-TrkA, 20-30 images were recorded with 10-second sampling intervals and 33 ms acquisition time. Single-molecule tracking of ATTO-488-p75^{NTR} and ATTO-633-TrkA was performed with custom-made software written in C++ (WinATR (Kusumi Lab, Membrane Cooperativity Unit, OIST, Japan). The center of each particle was localized by two-dimensional Gaussian fitting, and the trajectory for each signal was created by a minimum step size linking algorithm that connected the localized dots in subsequent images. The trajectories were individually checked, and artifacts or tracks shorter than 15

frames were excluded from further analysis. A minimum of 100 trajectories was collected in each experiment from neurites.

The specificity of the TrkA and $p75^{NTR}$ antibodies have been validated by preadsorption of the primary antibody with its corresponding fusion protein (blocking peptides: $TrkA_{342-356}$ and $p75^{NTR}_{188-203}$ peptides, Alomone Labs, Israel), and no immunoreactivity was observed (data not shown). To further test the specificity of TrkA and $p75^{NTR}$ antibodies Chinese hamster ovary (CHO) cells were transfected with plasmid encoding TrkA and $p75^{NTR}$ using Lipofectamine 3000 (CatN: L3000001; Thermo Fisher Scientific, Waltham, MA, USA) according to the manufacturer's protocol. Rat TrkA and rat $p75^{NTR}$ cDNA sequences were subcloned into a pHTC HaloTag® CMV-neo-Vector (Promega) expression vector under SgfI and PmeI sites. The TrkA and $p75^{NTR}$ cDNA sequences were a gift from Moses Chao (Addgene plasmid # 24093, Addgene plasmid # 24091; Watertown, MA, USA). The HaloTag constructs were verified with restriction analysis and Sanger sequencing. 24 hours after transfection cells were labeled and imaged in the same manner as detailed above. TrkA and $p75^{NTR}$ expressing CHO cells showed labeling with anti-TrkA ATTO-633 and anti-$p75^{NTR}$ ATTO-488 antibody, respectively, while native (non- TrkA, non- $p75^{NTR}$ expressing) CHO cells showed a complete absence of the signal (Supplementary Movie 4-5).

The viability of the cells was tested with a LIVE/DEAD viability/Cytotoxicity Assay Kit (CatN: L3224; Thermo Fisher Scientific, Waltham, MA, USA) at the end of experiments according to the manufacturer's instructions. The results demonstrated that cells retained their plasma membrane integrity until the end of the experiments (Supplementary Fig. 3.).

To verify whether labeled neurotrophin receptor molecules were moving on neurons, correlated live-cell single-molecule imaging and fixed cell immunocytochemistry were performed. After TrkA and $p75^{NTR}$ live-cell labeling and single-molecule tracking (described above), the glass-bottom dishes containing hiPSC neurons were fixed with 4% PFA in the

humidified chamber of the TIRF microscope. The x/y coordinates of at least 10-10 in vivo labeled TrkA and $p75^{NTR}$ positive neurites (ROI: 82 µm x 82 µm) were identified and saved using the Olympus Excellence Pro imaging software and x/y memory of the motorized microscope stage (Wetzlar, Germany). Cells were then fixed with 4% PFA and TUBB3 immunocytochemistry was performed under the TIRF microscope in the same manner as detailed above. Using the corresponding x/y coordinates and FIJI software [2] the movement of TrkA and $p75^{NTR}$ molecules were superimposed to TUBB3 positive images (Supplementary Movie 3).

Analysis of the diffusion parameters of TrkA and $p75^{NTR}$ molecules

Maximum likelihood estimation [3] was applied to obtain the corresponding diffusion coefficient for each trajectory. Δx_k and Δy_k represent the observed displacements ($\Delta x_k = x_{k+1} - x_k$ and $\Delta y_k = y_{k+1} - y_k$), arranged in N-component column vectors where the total number of frames is equal to N+1. x_n and y_n are the coordinates of the signal's center on the nth frame.

Σ is the N x N covariance matrix, defined by the following equation [4]:

$$\Sigma_{ij} = \begin{cases} 2D\Delta t - 2(2DR\Delta t - \sigma^2), & \text{if } i = j \\ 2DR\Delta t - \sigma^2, & \text{if } i = j \pm 1 \\ 0, & \text{otherwise} \end{cases}$$

where D is the diffusion coefficient, Δt is the frame integration time, σ is the static localization noise, R summarizes the motion blur effect and in our case R=1/6 because of the uniform illumination.

The likelihood function was defined by the following function:

$$L(\Delta x, \Delta y) = -log|\Sigma| - \frac{1}{2}(\Delta x)^T \Sigma^{-1}(\Delta x) - \frac{1}{2}(\Delta y)^T \Sigma^{-1}(\Delta y)$$

The D and σ which provide the maximal L (Δx, Δy) value is the estimated diffusion coefficient and static localization noise, respectively. This global optimization can be carried out directly. However, in this case it was necessary to calculate the determinant and the inverse of the covariance matrix at each step of the optimization method. In this case (the number of steps in a trajectory can be more than 200) this computational inconvenience becomes severe. Accordingly, we applied an approximation [5] which is based on the theory of circulant matrices, and it is applicable if the total number of frames is sufficiently high [4]. The global optimization was performed by a numerical method implemented in MATLAB. The goodness of optimization was judged by the evaluation of the static localization noise. An optimization was considered inaccurate and was excluded from further analysis when the estimated static localization noise was out of the ±90% range of the group's mean.

The presented MSD curves are the natural extension of the MSD curve of a single trajectory. We used the method which is applied in TrackArt [6]. We pooled all the *n* steps square displacements from all the trajectories in which the number of frames exceeds *n*, their mean represents the particular value of the MSD curve.

$$MSD(n\Delta t) = \frac{\sum_{i=1}^{m}\left(\sum_{j=1}^{N_i-n}\left(\left(x_{i,j+n} - x_{i,j}\right)^2 + \left(y_{i,j+n} - y_{i,j}\right)^2\right)\ [N_i > n]\right)}{\sum_{i=1}^{m}\left(\sum_{j=1}^{N_i-n} N_i - n\ [N_i > n]\right)}$$

where m is the number of the analyzed trajectories. N_i is the frame numbers in the ith trajectory, $x_{i,j}$ and $y_{i,j}$ are the coordinates at the jth frame of the ith trajectory while the expression in the bracket is the condition for the summation.

Clustering analysis of the D was performed with a custom-made software [7] implemented in LabView and successfully applied in the investigation of single-molecule diffusion-based estimation of ligand effects on receptors [8]. Briefly, the software provides a time series of diffusion states from the observed time series of trajectory coordinates. The

algorithm which computes the time series of diffusion states is based on the variational Bayes (VB) - hidden Markov model method with the assumption that the time series of trajectory coordinates obey the Markov process with some transition matrix. After the clustering analysis has been completed, the percentage of the slow, medium, and fast fractions of trajectories per neurites were calculated.

To determine the length of molecule trajectories, the distance between the detected localizations of the molecules was calculated by a MATLAB script for all two consecutive frames of each trajectory. The length of a molecule trajectory was defined as the sum of these distances and was normalized to steps (length-1). The area of TrkA and $p75^{NTR}$ molecule trajectories was defined as the area of the convex hull for each trajectory and was also normalized to steps.

Western blot

The samples of a representative *PSEN1* mutant and a non-demented individual (control) were used in the Western blot experiments. HiPSC-derived neuronal cultures were washed with PBS and lysed at 4°C in RIPA buffer containing protease and phosphatase inhibitors. After centrifugation (15.000 rpm, 15 min), supernatant was collected and stored at –80°C until use. The protein concentration was determined by the Bradford method. Denatured proteins were separated by sodium dodecyl sulfate (SDS) polyacrylamide gel electrophoresis, then electrically transferred onto nitrocellulose membranes (CatN: 1620115, Bio-Rad, Hercules, CA, USA). Membranes were first blocked with 5% nonfat dry milk in Tris-buffered saline/0.05% Tween-20 for 1 hour at RT. Membranes then were incubated with primary antibodies overnight at 4°C, followed by the appropriate HRP-conjugated secondary antibody (Table S4) for 2 hours at RT. Bands were visualized using SuperSignal West Pico chemiluminescent substrate (34080; Thermo Scientific, Waltham, MA, USA). Signals in each

lane were normalized to the loading control, GAPDH. Six samples of a control subject (Ctrl-2) and 6 samples of a fAD (fAD-1) patient were loaded repetitively and the mean value for each group was calculated using FIJI.

Statistical analysis

Dunnett's method was performed to compare the individual groups to controls in ELISA experiments. The Mann-Whitney U test was used to compare the diffusion area values, the trajectory length values, the diffusion coefficients, and the fractions of states. MSDs were compared with a two-sample t-test at each Δt. The diffusion coefficients measured at different time points of TrkA and $p75^{NTR}$ in control samples (10-minute intervals) were compared with Kruskal-Wallis ANOVA. Two-way repeated measure ANOVA was used when comparing the diffusion coefficients of TrkA and $p75^{NTR}$ every 10 minutes. As the measured diffusion coefficients did not change with time in any of the control samples, data from non-demented controls and fAD cell lines bearing *PSEN1* mutations were pooled and analyzed together when calculating all parameters. A two-sample t-test was applied to compare OD values of control and AD samples in the Western blot experiments. Normality and equal variance of the data were tested by Shapiro-Wilk test and F-test, respectively before applying the two-sample t-test.

Supplementary results

Aβ secretion by control and PSEN1 fAD hiPSC neurons

To validate the presence of Aβ in *PSEN1* fAD hiPSC neuron cultures, the secreted $A\beta_{1\text{-}40}$ and $A\beta_{1\text{-}42}$ levels were measured by ELISA in the conditioned media of the differentiated neural cells. Significantly elevated $A\beta_{1\text{-}40}$ and $A\beta_{1\text{-}42}$ levels and substantially increased $A\beta_{1\text{-}42}/A\beta_{1\text{-}40}$ ratios were detected in the *PSEN1* mutant cultures, compared to the non-demented hiPSC-

derived neurons, representing one of the major pathological phenotypes of AD (Supplementary Fig.1.).

Supplementary figures

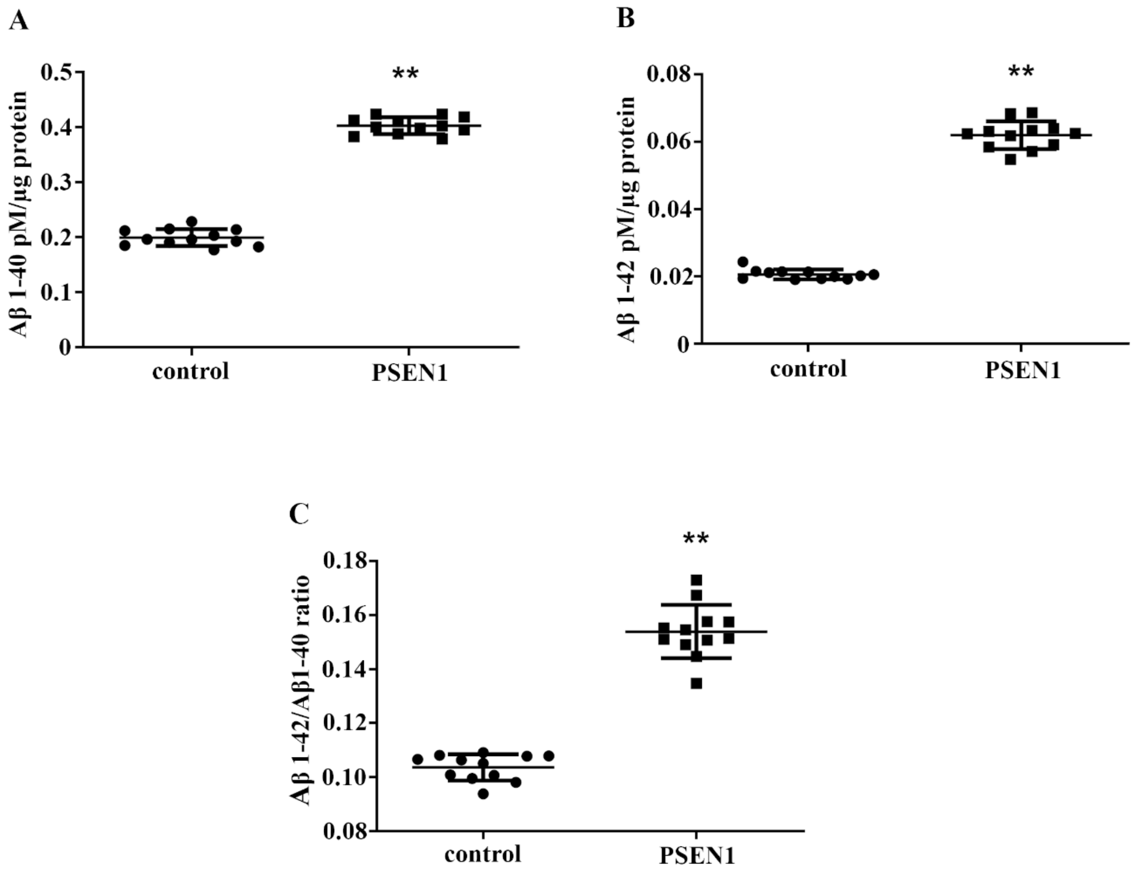

Figure S1. Levels of Aβ1-40 and Aβ1-42 in control and PSEN1 mutant neurons

Histograms show the levels of Aβ1-40, (A) Aβ1-42 (B) and the ratio of Aβ1-42/Aβ1-40 (C) in the media of control and *PSEN1* mutant neurons. The extracellular Aβ levels determined in pM were normalized to total protein content (in µg) of cell lysates. Data represent mean ± SEM (n=12). (**p<0.01).

Figure S2. Control staining with secondary antibodies

Representative image shows the background levels of signal when hiPSC neurons are labeled with Alexa Fluor 647 donkey anti-rabbit IgG. Scale bar = 10 µm.

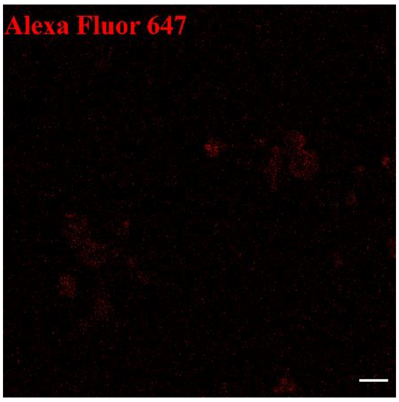

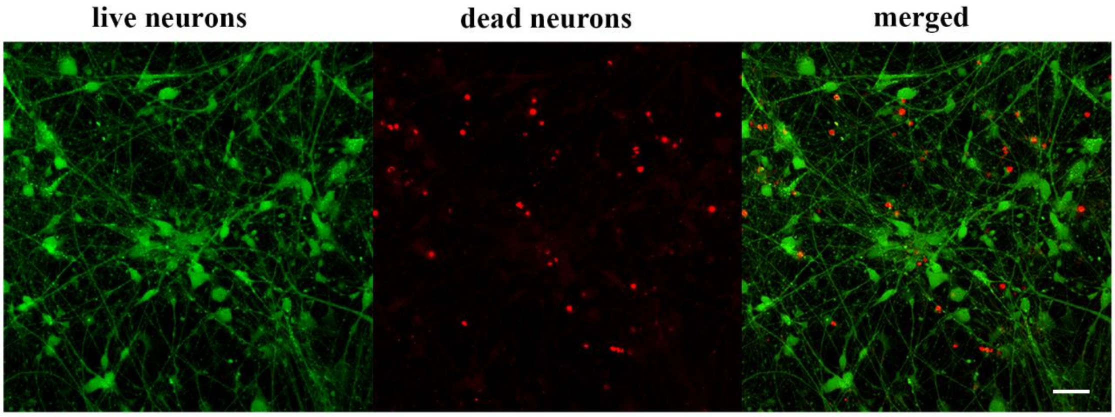

Figure S3. Viability of hiPSCs after measurements

Representative image shows that hiPSC neurons are alive after an hour measurement (A) Live cells are shown in green, dead cell population is labeled with red (green = calcein AM, red = ethidium homodimer-1). Scale bar = 50 µm.

Supplementary tables

Table S1. Diffusion parameters of TrkA and $p75^{NTR}$ in hiPSC-derived control and fAD neurons

Sample	TrkA		p75NTR	
**Parameter**	**control**	**fAD**	**control**	**fAD**
**D ($\mu m^2/s$)**	$0.2644 \pm 1.42*10^{-2}$	$0.2446 \pm 1.23*10^{-2}$	$0.299 \pm 8.39*10^{-3}$	$0.2411 \pm 5.88*10^{-3}$
**area (μm^2/step)**	$0.0127 \pm 4.37*10^{-4}$	$0.0107 \pm 3.81*10^{-4}$	$0.0143 \pm 2.84*10^{-4}$	$0.0112 \pm 2*10^{-4}$
**trajectory length (µm/step)**	$0.2036 \pm 2.43*10^{-3}$	$0.1777 \pm 2.05*10^{-3}$	$0.2199 \pm 1.56*10^{-3}$	$0.1954 \pm 1.28*10^{-3}$

The table shows the diffusion coefficients, the diffusion area, and the trajectory length of TrkA and $p75^{NTR}$ molecules in hiPSC-derived control and fAD neurons. Data are expressed as mean ± SEM.

Table S2. Diffusion states of TrkA and $p75^{NTR}$ in hiPSC-derived control and fAD neurons

Sample	TrkA control		TrkA fAD		p75NTR control		p75NTR fAD	
**Fractions**	**percentage**	**D**	**percentage**	**D**	**percentage**	**D**	**percentage**	**D**
**slow**	23.46%	$0.0356 \pm 2.9*10^{-3}$	28.68%	$0.0248 \pm 1.76*10^{-2}$	17.58%	$0.0712 \pm 6.32*10^{-3}$	31.90%	$0.0554 \pm 3.85*10^{-3}$
**medium**	46.24%	$0.0952 \pm 6.66*10^{-3}$	44.87%	$0.1303 \pm 1.15*10^{-2}$	48.67%	$0.1571 \pm 6.99*10^{-3}$	46.17%	$0.1548 \pm 6.58*10^{-3}$
**fast**	30.30%	0.7413 ± 6.8310^{-2}	26.45%	$0.7581 \pm 4.36*10^{-2}$	33.74%	$0.6323 \pm 2.47*10^{-2}$	21.93%	$0.6546 \pm 2.86*10^{-2}$

The table shows the percentage of slow, medium, and fast fraction of TrkA and $p75^{NTR}$ molecules in hiPSC-derived control and fAD neurons. Data are expressed as mean ± SEM.

Table S3. Antibodies used for immunocytochemistry

	Antibody	Dilution	Company (Cat #)
**Neuronal differentiation markers**	Rabbit anti-MAP2	1:1000	Millipore (MAB3418)
**Neuronal phenotype markers**	Mouse anti-TUBB3<br>Rabbit anti-VGLUT 1/2<br>Rabbit anti-GAD65/67<br>Rabbit anti-VAChT<br>Rabbit anti-TrkA<br>Mouse anti-P75^{NTR}	1:500<br>1:500<br>1:1000<br>1:1000<br>1:100<br>1:1000	Santa Cruz (sc-58888)<br>Synaptic Systems (135503)<br>Abcam (ab49832)<br>Synaptic Systems (138103)<br>Abcam (ab76291)<br>Biolegend (345101)
**Secondary antibodies**	Alexa Fluor 488 donkey anti-rabbit IgG<br>Alexa Fluor 594 donkey anti-mouse IgG<br>Alexa Fluor 647 donkey anti-rabbit IgG<br>Alexa Fluor 647 F(ab')$_2$ donkey anti-mouse IgG	1:2000<br>1:2000<br>1:2000<br>1:2000	Thermo Fisher (A-21206)<br>Thermo Fisher (A-21203)<br>Jackson (711-605-152)<br>Jackson (715-606-150)

Table S4. Antibodies used for Western blot

	Antibody	Dilution	Company (Cat #)
**Primary antibodies**	Rabbit anti-TrkA<br>Rabbit anti- P75^{NTR}<br>Rabbit anti-ERK 1/2<br>Rabbit anti-phospho-ERK1/2<br>Rabbit anti-Akt<br>Rabbit anti-phospho-Akt<br>Rabbit anti-SAPK/JNK<br>Rabbit anti-phospho-SAPK/JNK<br>Rabbit anti-GAPDH	1:1000<br>1:500<br>1:1000<br>1:1000<br>1:1000<br>1:1000<br>1:1000<br>1:1000<br>1:10000	Santa Cruz (sc-11)<br>Alomone (ANT-007)<br>Cell Signaling (4695)<br>Cell Signaling (9101)<br>Cell Signaling (9272)<br>Cell Signaling (9271)<br>Cell Signaling (9252)<br>Cell Signaling (9251)<br>Alomone (ab181603)
**Secondary antibodies**	Goat anti-rabbit IgG HRP Conjugate	1:2500	Promega (W4011)

Supplementary movies

Movie S1. Surface movements of TrkA in control hiPSCs

Single molecules of ATTO-633-labeled TrkA are moving along a neurite in live hiPSC-derived neuron from a non-demented individual.

Movie S2. Surface movements of $p75^{NTR}$ in control hiPSCs

Single molecules of ATTO-488-labeled $p75^{NTR}$ are moving along a neurite in live hiPSC-derived neuron from a non-demented individual.

Movie S3. Correlated live-cell single-molecule imaging and fixed cell immunocytochemistry

Representative video shows that ATTO-488-labeled $p75^{NTR}$ molecules (green) are moving along a beta-III tubulin immunopositive neurite (red).

Movie S4. ATTO-633-labeled TrkA molecules moving on the membrane of a live transfected CHO cell

Movie S5. ATTO-488-labeled $p75^{NTR}$ molecules moving on the membrane of a live transfected CHO cell

Supplementary references

[1] Chambers SM, Fasano CA, Papapetrou EP, Tomishima M, Sadelain M, Studer L. Highly efficient neural conversion of human ES and iPS cells by dual inhibition of SMAD signaling. Nat Biotechnol 2009;27:275–80.

[2] Schindelin J, Arganda-Carreras I, Frise E, Kaynig V, Longair M, Pietzsch T, et al. Fiji: an open-source platform for biological-image analysis. Nat Methods 2012;9:676–82.

[3] Zwiernik P, Uhler C, Richards D. Maximum likelihood estimation for linear Gaussian covariance models. J R Stat Soc Ser B (Statistical Methodol 2017;79:1269–92.

[4] Berglund AJ. Statistics of camera-based single-particle tracking. Phys Rev E 2010;82:11917.

[5] Gray RM. Toeplitz and Circulant Matrices: A Review. Found Trends® Commun Inf Theory 2006;2:155–239.

[6] Matysik A, Kraut RS. TrackArt: the user friendly interface for single molecule tracking data analysis and simulation applied to complex diffusion in mica supported lipid bilayers. BMC Res Notes 2014;7:274.

[7] Hiroshima M, Pack C gi, Kaizu K, Takahashi K, Ueda M, Sako Y. Transient Acceleration of Epidermal Growth Factor Receptor Dynamics Produces Higher-Order Signaling Clusters. J Mol Biol 2018;430:1386–401.

[8] Yanagawa M, Hiroshima M, Togashi Y, Abe M, Yamashita T, Shichida Y, et al. Single-molecule diffusion-based estimation of ligand effects on G protein–coupled receptors. Sci Signal 2018;11:eaao1917.
